# Microchemical system for simultaneous measurement of surface-enhanced Raman scattering and electrochemical reactions

**DOI:** 10.1038/s41598-025-02647-y

**Published:** 2025-05-27

**Authors:** Shunya Saegusa, Masayuki Naya, Takao Fukuoka, Miyuki Tabata, Koji Sumitomo, Akinobu Yamaguchi

**Affiliations:** 1 Laboratory of Advanced Science and Technology for Industry, University of Hyogo, Ako-gun, Hyogo 678-1205 Japan; 2 Faculty of Science and Technology, Keio University, Yokohama, 223-0061 Japan; 3 Department of Micro Engineering, Kyoto University, Kyoto Daigaku-Katsura, Nishikyo-ku, Kyoto, 615- 8540 Japan; 4 Graduate School of Bio-Applications and Systems Engineering, Tokyo University of Agriculture and Technology, Naka-cho, Koganei, Tokyo, 184-8588 Japan; 5 Graduate School of Engineering, University of Hyogo, Shosha, Himeji, Hyogo 671-2280 Japan; 6 Department of Electrical, Electronic and Communications Engineering, Faculty of Science and Engineering, Toyo University, 2100 Kujirai, Kawagoe, Saitama 350-8585 Japan

**Keywords:** Chemistry, Surface chemistry, SERS, Chemical engineering

## Abstract

Electrochemical reactions have been extensively studied and used in various fundamental research and engineering applications such as electroplating, surface treatment, secondary batteries, and fuel cells because they are scalable, economically viable, and practical processes. Research on electrochemical reactions is ongoing because they still contain unknown phenomena. In particular, the ability to conduct reactions at interfaces while precisely controlling non-equilibrium chemical reaction states electrically contributes to the analysis of reaction mechanisms and industrial applications. In this study, we fabricated a gold nanofève structure as a working electrode and traced the chemical reaction processes during electrochemical reactions by simultaneous measurement of surface-enhanced Raman scattering (SERS) spectra. In addition to confirming the reattachment of 4-mercaptobenzoic acid by electrochemical manipulation, the redox reactions and formation of copper nanoparticles in aqueous copper acetate solutions were tracked by in situ SERS measurements while controlling electrochemical reactions. This enabled in situ real-time SERS measurements on the surface of the working electrode in the microsystem during electrochemical reactions. The developed system can be used to measure non-equilibrium chemical reaction dynamics at solid–liquid interfaces to explore related phenomena.

**Supplementary Information:**

The online version contains supplementary material available at 10.1038/s41598-025-02647-y.

## Introduction

Electrochemistry has been studied extensively and is applied in a wide range of fields including analysis, where it is used to observe oxidation and reduction reactions, and industrial chemistry, where it is used for reaction processes such as electroplating^1,2^ In materials science, electrochemistry contributes to electroforming and batteries, whereas in biotechnology, electrochemistry aids the elucidation of neurotransmission and photosynthesis. Electrochemical reactions occur in the electrode bilayer within a few micrometers of the electrode surface, so it is important to track molecules at the interface between the solid electrode and liquid electrolyte. Understanding nanoscale electrochemistry at heterogeneous interfaces and on electrode surfaces is important in fundamental science and for elucidating control mechanisms in engineering applications. Recent developments in the nanofabrication of electrode materials for applications including sensing and electrocatalysis have revealed that nanoscale features on the electrode surface and a heterogeneous interface between electrode and electrolyte affect the mechanism, kinetics, and thermodynamics of interfacial redox reactions^3 – 21^.

The most common techniques to map spatially varying electrochemistry are scanning electrochemical microscopy,^22^ scanning ion-conductance microscopy,^23^ and in situ photon-in/photon-out fluorescence-yield X-ray absorption spectroscopy using synchrotron radiation^10 – 12^ The spatial resolution of both scanning electrochemical and scanning ion-conductance microscopy is limited by the tip geometry and diffusion. In fluorescence-yield X-ray absorption spectroscopy, the spatial resolution is limited by the radius of the X-ray beam focused using a Fresnel zone plate. The former techniques acquire electrochemical current, whereas the latter measures the characteristic fluorescence X-ray energy during an electrochemical reaction. Vibrational spectroscopies such as surface-enhanced Raman scattering (SERS),^24 – 26^ tip-enhanced Raman spectroscopy (TERS),^13,25 – 30^ and infrared absorption spectroscopy^26^ can provide information about interfacial chemical properties. Recently, both electrochemical surface-enhanced Raman scattering (EC-SERS)^7 – 9,17,18^ and electrochemical tip-enhanced Raman spectroscopy (EC-TERS)^3,4,13 – 16^ have attracted attention because they can reveal the mechanisms of electrochemical adsorption and reactions at the interfaces and surfaces of electrodes. In both EC-SERS and EC-TERS, the potential of the electrode can be actively manipulated so that the Fermi level of the electrode can be modulated to excite resonance transitions to the highest occupied or lowest unoccupied molecular orbital of a molecule trapped in a nanogap or to shift resonant conditions. With TERS, the use of a scanning probe allows such manipulation to be locally limited at the atomic level, and the chemical state of a single molecule can be detected in Raman spectra while arbitrarily setting the desired location and controlling the potential. Therefore, many studies using EC-TERS have been reported. For example, Kang et al.. used in situ electrochemical atomic force microscopy tip-enhanced Raman spectroscopy (EC-AFM-TERS) to spatially resolve local heterogeneity in the redox behavior on an electrode surface^29^ They monitored the TERS intensity of Nile blue molecules adsorbed on the electrode surface and obtained TERS maps with different applied potentials. Such in situ redox mapping at the nanoscale using EC-AFM-TERS advances understanding of the effect of nanoscale surface features on chemical reactions. Recently, Fiocco *et al.*. demonstrated a real-time EC-STM-TERS configuration to investigate complex electrochemical reaction at modified electrode surfaces^30^ Their approach was successfully applied to the investigation of a well-established but yet complex system associated with a thiolated nitrobenzene derivative 4-nitrobenzyl mercaptan (4-NBM) whose reduction mechanism involves various multistep reaction paths, also being dependent on pH.

Although both EC-AFM-TERS and EC-STM-TERS can provide detailed information, it requires expensive equipment^27 – 30^ In addition, the time resolution of both is lowered when imaging is performed. However, even in EC-SERS, when scanning imaging is performed using micro-Raman spectroscopy, it is inevitable that the time resolution will be lower than when the probe is fixed at a single location. With the use of a modern hyperspectral camera, it is possible to achieve both sufficient temporal resolution, albeit at the expense of spatial resolution to some extent, and mapping. In this respect, EC-SERS is also an effective method for investigating chemical reactions at heterogeneous interfaces and surfaces because it allows molecular spectral tracking using SERS activity. Integration of an electrochemical cell with a SERS-active electrode in a microchemical system^31 – 37^ will provide a platform to investigate electrochemical reaction dynamics at electrode surfaces and solid–liquid interfaces.

In this study, we implement EC-SERS in a microchemical system for ease of use. There are various methods to fabricate SERS-active structures, such as nanoimprinting, colloidal aggregation, and reduction of metal ions. Here, we fabricate a SERS-active gold nano coral (GNC) structure on a boehmite substrate because it is suitable for high-sensitivity and high-resolution SERS measurements over a relatively wide area^37 – 40^ The SERS sensitivity is dependent on the GNC structure, and previous studies have provided insight into the fabrication of the optimum GNC structure with adequate high SERS activity^37,40^ The GNC patterns are fabricated by magnetron sputtering to allow integration with a microfluidic system^37,40^ The resulting system is easy to fabricate on a microchip and can achieve in situ EC-SERS with high resolution and sensitivity. The system can be incorporated into a micro-Raman microscope or used in combination with a mobile Raman spectrometer for field work. As a performance test of the system, the redox reaction process of copper is simultaneously observed by cyclic voltammetry (CV) and Raman spectroscopy.

In addition, 4-mercaptobenzoic acid is attached to the GNC surface to evaluate the ability of the system to detect changes in reactions caused by different surface conditions. Mercaptobenzoic acid has thiol groups and is well known to bind to gold surfaces. Therefore, we wondered whether, in electrochemical reactions on aqueous copper acetate solutions at gold nanostructured electrodes, there would be competition in chemical reactions at the electrode/liquid interface, as in competitive Enzyme-Linked Immunosorbent Assay (ELISA), in redox reactions when mercaptobenzoic acid is adsorbed on the gold nanostructured electrode surface and when it is not. The study was conducted in order to investigate the possibility of competition in redox reactions. Therefore, in this study, the electrochemical reactions were investigated using gold nanostructured electrodes with and without mercaptobenzoic acid.

## Methods and materials

### Electrode and device fabrication

**Fig. 1:**
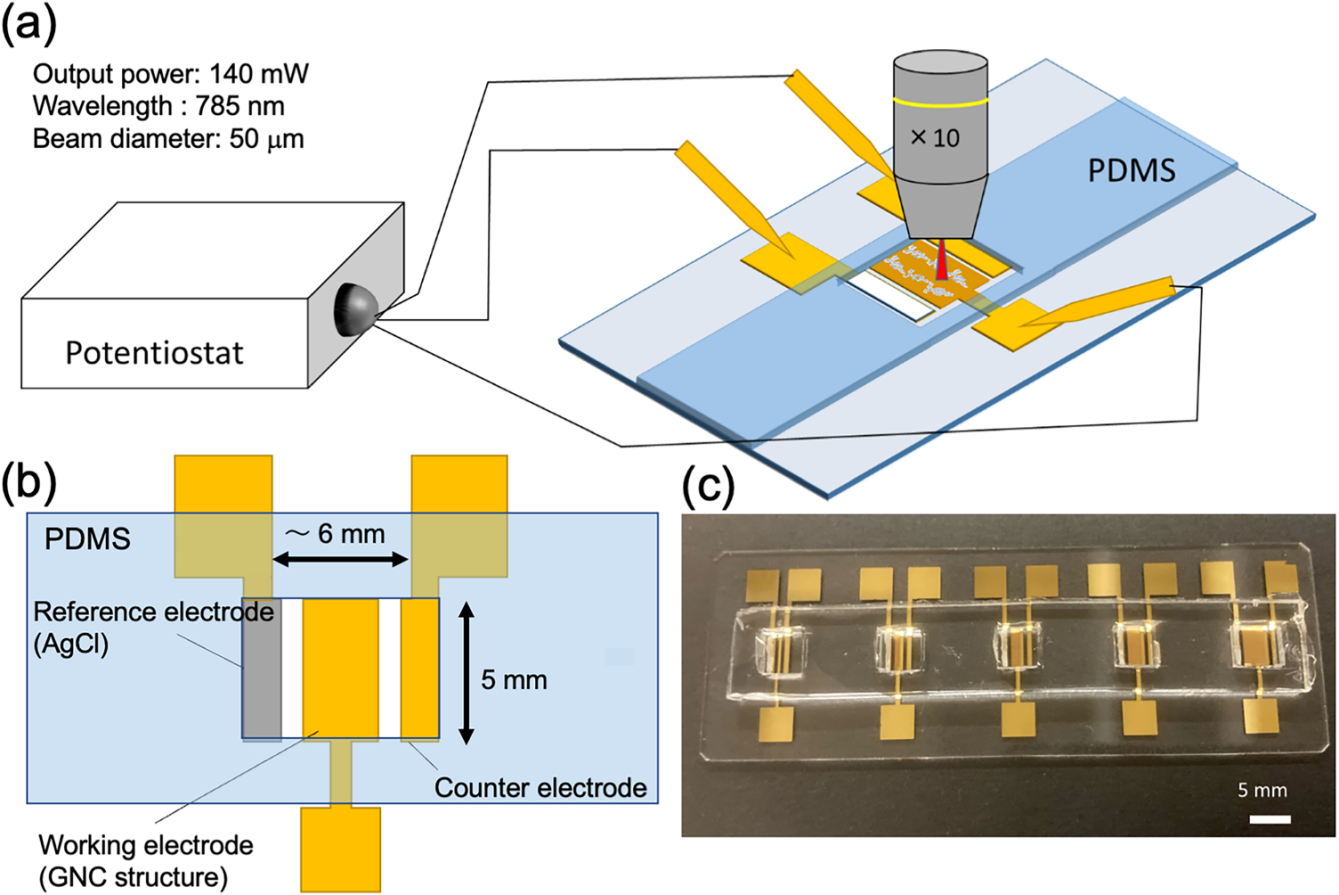
(**a**) Schematic diagram of the setup for surface-enhanced Raman scattering (SERS) spectroscopy measurements during electrochemical reactions using the microchemical system. (**b**) Schematic diagram of the microchemical system. (**c**) Photograph of the microchemical system.

Figure 1a shows the basic structural arrangement of the electrodes and a schematic diagram of the system and measurement setup containing poly(dimethylsiloxane) (PDMS) cells^37,41^ The electrochemical reaction cell was made of PDMS by soft mold lithography technique. Figure 1b and c show a schematic diagram and optical photograph of the fabricated system, respectively. The central electrode is the working electrode, which has a SERS-active GNC structure that was fabricated by sputtering Au on a boehmite aluminum hydroxide substrate with a needle-like structure. The GNC structure was fabricated by growing Au nanoparticles from the sharp end of the boehmite needles by vapor deposition or sputtering^37,40^ The reference electrode consisted of Ag/AgCl pasted on a gold electrode. The counter electrode was a standard Au electrode.

**Fig. 2:**
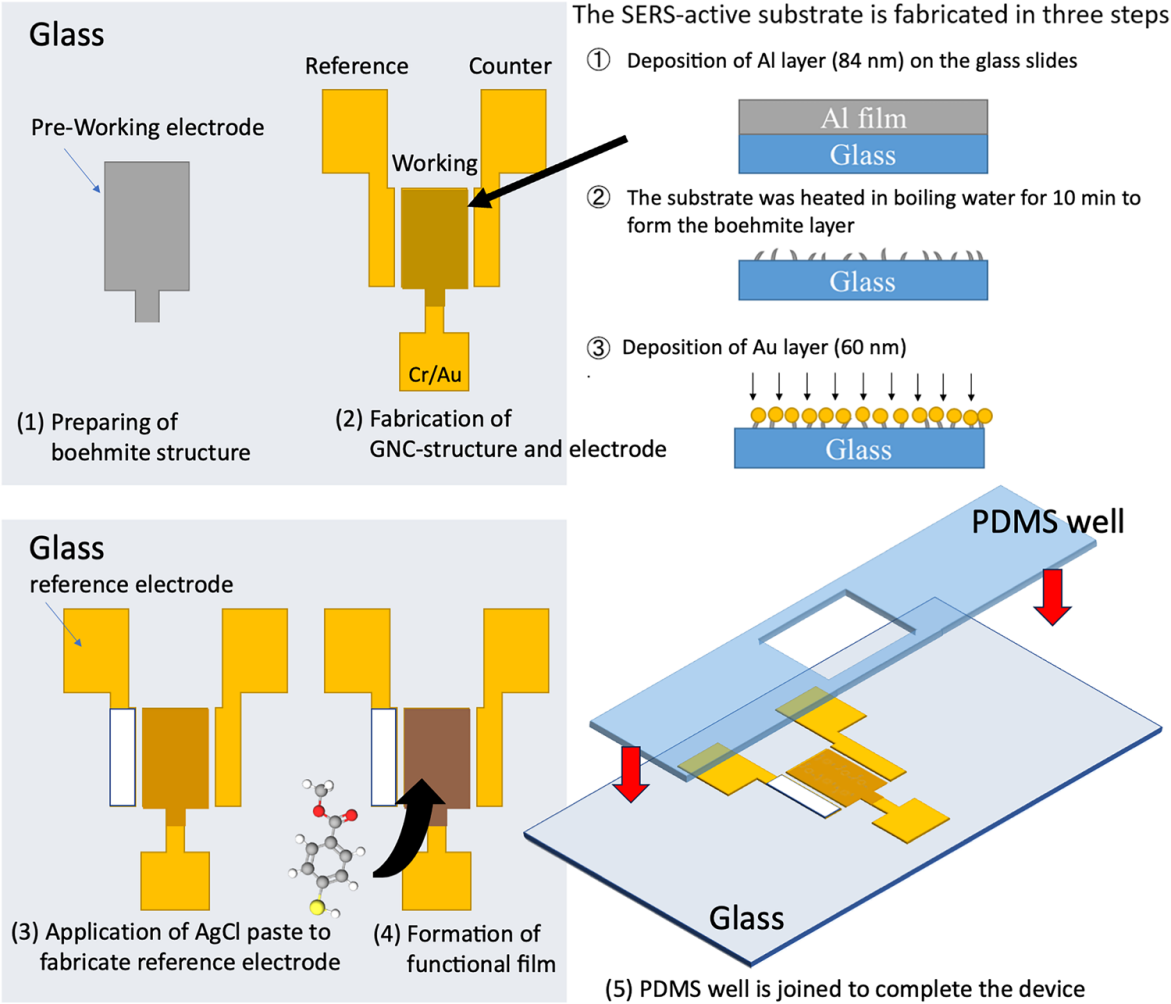
Schematic diagrams of the microchip fabrication process and formation of a gold nano coral (GNC) structure on the working electrode. Fabrication was conducted from step (1) to (5) in order. Step (4) was only included if a surface atomic monolayer (SAM) was required on the working electrode.

The fabrication process is schematically depicted in Fig. 2. First, the working electrode was formed by sputtering Cr (10 nm) and Al (84 nm) through a metal mask onto a glass slide previously cleaned with acetone, isopropanol, and pure water. For pattering, sputtering deposition was carried out using a metal mask with the geometry shown in Fig. 2. A boehmite structure was formed by immersing the substrate in boiling pure water for 10 min. After drying the substrate, it was sputtered with Cr (10 nm) and Au (180 nm). The electrode was coated with Ag/AgCl paste and then heated at 100 °C for 5 min using a hot plate. The fabricated electrode structure is shown in Figs. 1 and 2. The center of the electrode structure with dimensions of 5 × 1.5 mm was used as the working electrode and the counter and reference electrodes had dimensions of 5 × 1 mm. Depending on the objective of the investigation, a functional film could then be deposited on the surface of the GNC electrode, as schematically illustrated in Fig. 2. After the formation of a functional film on the GNC electrode, it was attached to a PDMS microcell to complete the system. As for uncertainty, this may be caused by variations in the Boehmite structure or sputter deposition, or by the adsorption of some kind of contaminant on the SERS structure. However, Ultra Violet light irradiation treatment was carried out before the electrochemical measurements were carried out, and the SERS electrode was confirmed to be functioning normally by carrying out the electrochemical measurements and confirming a clear CV before carrying out the experiments.40 µL of analyte solution was filled in the cell. The system was connected to a potentiostat (EC stat-302, EC Frontier Co. Ltd., Japan) and positioned on a micro-Raman spectrometer (Fig. 1).

### Operando SERS spectroscopy during CV measurements

Operando in situ EC-SERS was performed with a customized Raman spectrometer system (Nikon FN-1 microscope with a Lambda Vision RAMS300S spectrometer; excitation wavelength: 785 nm, laser power: 140 mW, 10× objective lens, beam diameter: ~50 μm). The SERS measurements during the electrochemical measurements were conducted at room temperature in the device depicted schematically in Fig. 1. We focused on the chemical reactions at the interface between the GNF electrode and liquid electrolyte. Potentials were converted to the reversible hydrogen electrode (RHE) scale using the equation $\:{E}_{\text{R}\text{H}\text{E}}=\:{E}_{\text{A}\text{g}|\text{A}\text{g}\text{C}\text{l}}$ + 0.205 V + 0.059 V × pH. The pH of the 10 mM copper(II) acetate (Cu(OAc)_2_, where AcO^−^ is an acetate ion (CH_3_COO^−^)) electrolyte was 5.4. The collection time of each spectrum depended on the electrochemical protocol. In this study, the collection time was set to 50 ms. The CV was measured at an initial potential of 0.023 V and a scan rate of 0.02 V/s.

## Result and discussion

In this study, we performed two experiments. First, the operando in situ EC-SERS of 10 mM Cu(OAc)_2_ solution only was measured to investigate the chemical reactions induced on the GNC surface. Second, to study the characteristics of a surface atomic monolayer (SAM) on the GNC electrode at the interface between the solid GNC electrode and liquid Cu(OAc)_2_ electrolyte, we conducted operando in situ EC-SERS measurements after 4-mercaptobenzoic acid was coated as a SAM on the GNC electrode surface. EC-SERS measurements of the mercaptobenzoic acid solution only were reported in the supplementary information^42^.

In the first experiment, CV of Cu(OAc)_2_ solution was measured to confirm that the system could be used to observe chemical reaction processes. The following chemical reactions can occur in the electrochemical system^43,44^

1 $${\text{C}}{{\text{u}}^{{\text{2}}+}}~+~~{\text{2}}{{\text{e}}^ - }~ \to {\text{Cu}},$$

2 $${\text{C}}{{\text{u}}^+}~+~~{{\text{e}}^ - }~~ \to {\text{Cu}},$$

3 $${\text{4Cu }}+~{{\text{O}}_{\text{2}}} \to {\text{2C}}{{\text{u}}_{\text{2}}}{\text{O}},$$

4 $${\text{C}}{{\text{u}}_{\text{2}}}{\text{O }}+\frac{1}{2}{{\text{O}}_{\text{2}}} \to {\text{CuO}},$$

5 $${\text{Cu}}\,+\,{\text{Cu}}{\left( {{\text{OAc}}} \right)_{\text{2}}} \to {\text{2Cu}}\left( {{\text{OAc}}} \right).$$

Other reactions may also occur. Cu(OAc)_2_ can form monomers and dimers, and is not well understood in some respects. Here, we list examples that have been reported and reactions that can easily be imagined. The following typical reduction processes of Cu can be also induced by electrochemical reactions:

6 $${\text{2}}{{\text{H}}^+}+{\text{ 2CuO}} \to {\text{C}}{{\text{u}}_{\text{2}}}{\text{O}}\,+\,{{\text{H}}_{\text{2}}}{\text{O}},$$

7 $${\text{2}}{{\text{H}}^+}+{\text{C}}{{\text{u}}_{\text{2}}}{\text{O}} \to {\text{2C}}{{\text{u}}^{{\text{2}}+}}+{\text{ }}{{\text{H}}_{\text{2}}}{\text{O}}.$$

These redox reactions can be actively controlled by electrochemical reactions using the GNC electrode in the microchemical system.

**Fig. 3:**
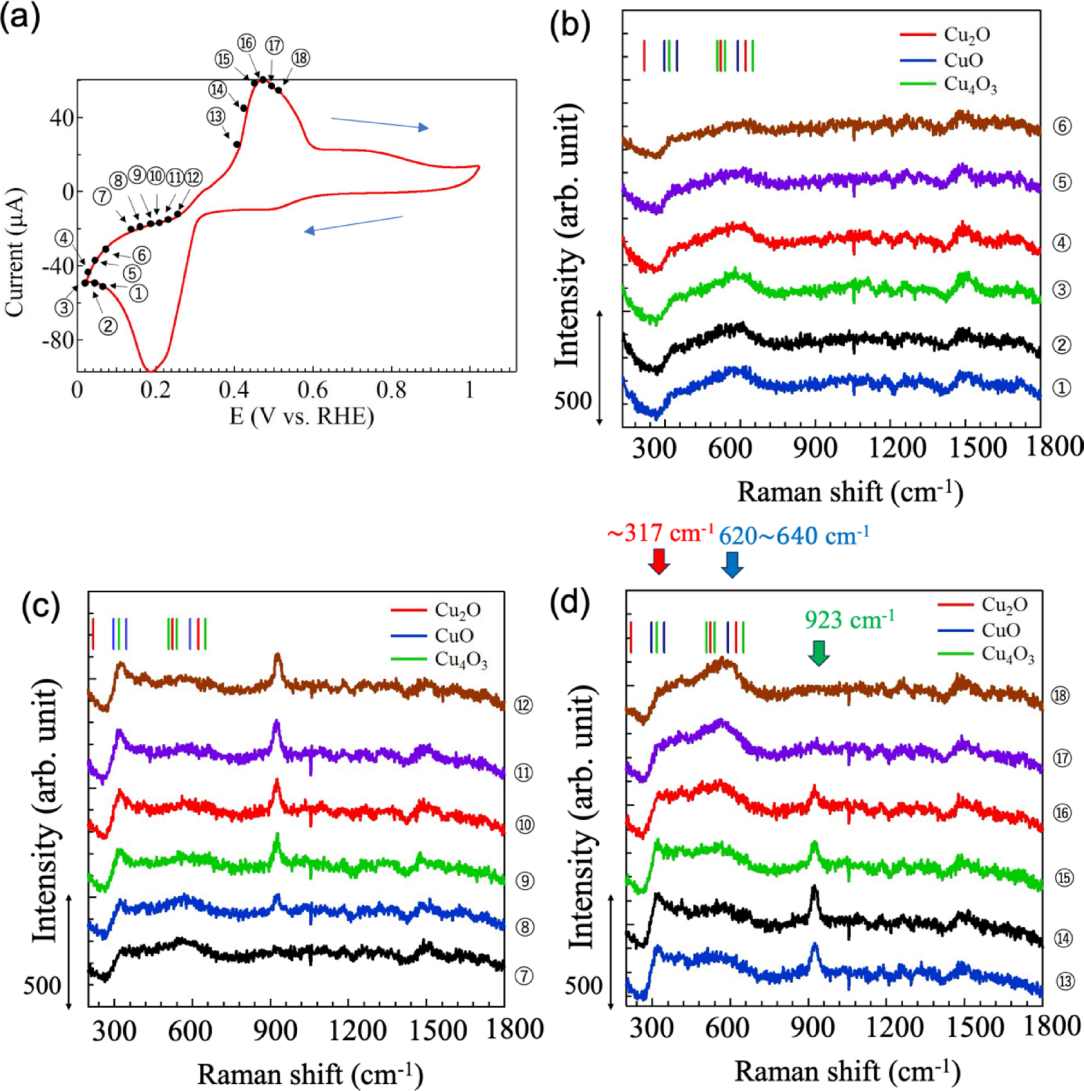
(**a**) Cyclic voltammetry (CV) curves of a GNC electrode immersed in 10 mM copper acetate liquid electrolyte in the microchemical system. Numbers correspond to the measurement points of the SERS spectra. (**b**–**d**) SERS spectra collected at each measurement point. For clarity, spectra are shifted vertically.

Figure 3a shows the CV curve for Cu(OAc)_2_ only without any additives. The curve shows an oxidation peak at 0.48 V and reduction peak at 0.20 V. The SERS spectra of the GNF electrode surface measured at the locations of point (1)–(18) on the CV curve in Fig. 3a are shown in Fig. 3b–d. Figure 3b displays SERS spectra collected at point (1)–(6). For clarity, each spectrum is vertically shifted. Comparing the spectra reveals that they are similar. The SERS spectra contain a broad peak between 300 and 650 cm^− 1^, and the intensity of this peak appears to decrease slightly from point (1) to (6) in Fig. 3b. These Raman signals originated from the copper oxides and hydroxides produced by electrochemical reactions. The coloured vertical lines indicate the Raman shift positions where the characteristic Raman peaks of (red) Cu_2_O, (blue) CuO and (green) Cu_4_O_3_ appear, respectively. The signal intensity clearly varied with increasing RHE voltage from point (7) to (12), as shown in Fig. 3c. The intensity of the peak at about 317 cm^− 1^, which was derived from CuO, increased with RHE voltage. Simultaneously, the Raman peaks around 600–640 cm^− 1^ broadened and their intensity weakened. A peak at 923 cm^− 1^ appeared abruptly at point (7) and strengthened as the RHE voltage increased from point (7) to (14) in Fig. 3d. After point (15), the Raman peak at 923 cm^− 1^ gradually decreased in intensity with the further increase of RHE voltage. Similarly, the Raman peak intensity at 317 cm^− 1^ also decreased with increasing RHE voltage from point (15) to (18). In contrast, the Raman peak intensity around 600–640 cm^− 1^ increased with RHE from point (15) to (18), as shown in Fig. 3d. Here, the Raman peaks at 317 and around 600–640 cm^− 1^ in Fig. 3b–d originated from the Raman modes of CuO and Cu_2_O, respectively^45,46^ The peak observed at 923 cm^− 1^ was assigned to the C-C νC-C/δCO_2_ peak, that is acetate ions^44^.

These observed changes in the SERS spectra during the electrochemical measurements can be explained as follows. First, Cu was reduced at the surface of the GNC electrode, then, from point (1) to (7) on the CV curve in Fig. 3a, a Cu_2_O layer nucleated and gradually grew on the GNC surface. At this time, Cu ions on or near the GNF electrode were expected to be involved in the formation of the Cu_2_O layer. When the electrochemical reaction proceeded to point (8), Cu_2_O began to oxidize to CuO, as indicated by the increasing intensity of the broad overlapping CuO and Cu_2_O peaks around 570 cm^− 1^. This transition may be caused by the progressive oxidation of CuO, which increases the volume of the surface layer on the electrode, thus decreasing the nanogaps between GNF particles and weakening the sensitivity of SERS detection. When copper ions are reduced at the GNC electrode from the aqueous Cu(OAc)_2_ solution, AcO^−^ are also entrapped at the GNC electrode, and thus an acetate-induced peak was considered to be observed. This means that the electrochemical reaction progress at the electrode–electrolyte interface was detected label-free in situ. In addition, copper oxides are also considered to have formed and are easily detectable. The oxidation reaction occurred until point (15). When the electrochemical reaction was in progress from the point (15) to the point (18), the decrease in current in the CV. The corresponding Raman spectra in Fig. 3d shows that the peak at 923 cm^− 1^ attributed to acetate ions has decreased and disappeared from point (15) to point (18). This indicates that the acetate ions adsorbed on the electrode surface may have eluted into the liquid. At the same time, the peak around 317 cm^− 1^ decreases and the broad peak around 600–640 cm^− 1^ increases. This may indicate a decrease in CuO-derived components and an increase in Cu_2_O-derived components. In other words, it is inferred that mass transfer effects are also occurring while the associated redox reactions are being excited. In the CV process, Cu and acetate ions were adsorbed on the GNC electrode and SERS was observed, suggesting that the oxygen from acetate ions facilitated oxidation of copper to promote nucleation and crystal growth simultaneously. Operando SERS observations during electrochemical reaction excitation with a GNC-SERS-active structure electrode show that such reaction dynamics can be detected and monitored in real-time for the first time.

**Fig. 4:**
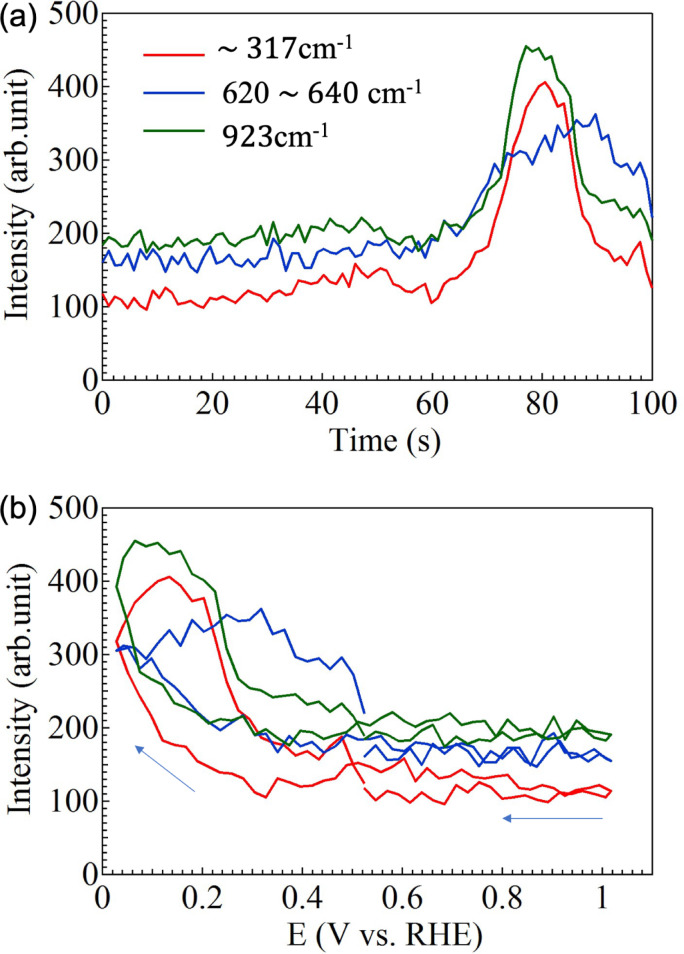
(**a**) CV time and (**b**) RHE voltage dependence of Raman peaks at $\:\sim$317 620 $\:\sim$ 640, and 923 cm^−1^.

After the oxidation process was complete, the reduction process from CuO to Cu_2_O continues to proceed as described above. The oxidation and reduction processes show the same trend in Fig. 4a and b, which depict the dependence of the Raman peaks derived from Cu_4_O_3_ (~ 320 cm^− 1^, E_g_ mode) or CuO (~ 320 cm^− 1^, A_g_ mode), Cu_2_O (620$\:\sim$640 cm^−1^, Bg mode), and C-C, νC-C/δCO_2_ ($\:\sim$923 cm^− 1^)^45, 46^. The results shown in Fig. 4a and b show that the Raman peaks originating from CuO and COO occur at the same timing and at the same RHE voltage. The Raman peak originating from Cu_2_O also appears almost at the same timing and the RHE voltage dependence of the Raman peaks seems to be a little different from that of the other CuO and COO peaks. This may indicate that copper acetate precipitates as CuO together with COO on the GNC electrode surface and is reduced to Cu_2_O by electrochemical reaction^43,44^.

Considering the EC-SERS measurements further, an oxygen atom of the acetate ion is drawn to the electrode, which is thought to have specifically increased the peak intensity at 923 cm^− 1^. These species are believed to have been attracted to the electrode and attached to the hot spots provided by the GNC structure, enabling their SERS detection. The deposition of copper may also have generated new hot spots and contributed to the high SERS sensitivity of the system. In addition, the copper oxidation process can be traced in detail during the electrochemical reaction. Raman measurements tracking the electrochemical reaction could not be observed on a flat Au electrode without a SERS structure. Overall, our EC-SERS system allows SERS measurement and chemical reaction tracing of the electrochemical reaction process at lower concentrations than was previously possible.

**Fig. 5:**
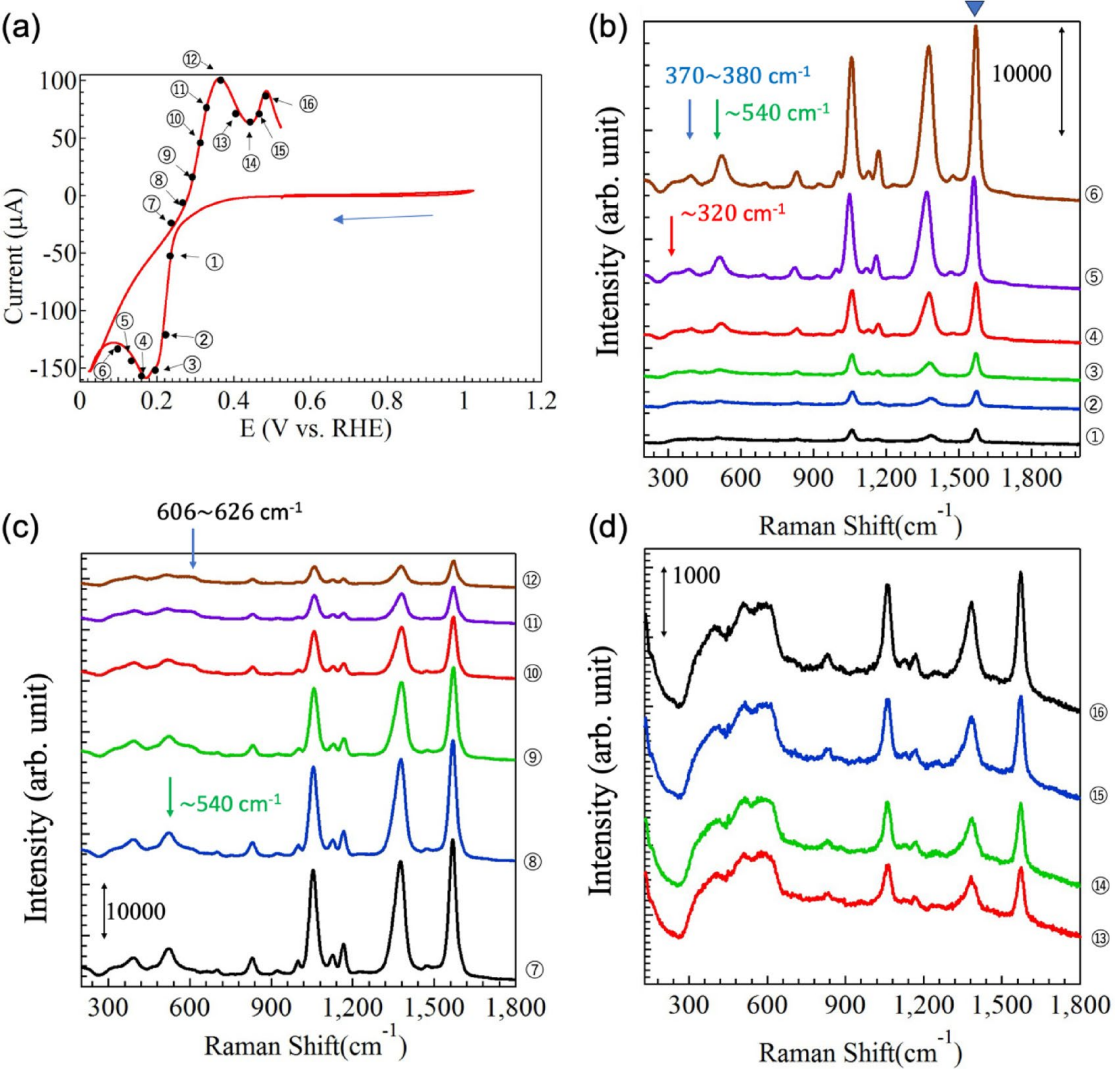
(**a**) CV curve of a GNC electrode coated with 4-mercaptobenzonic acid as a SAM and immersed in 10 mM copper acetate electrolyte. Numbers correspond to the measurement points of SERS spectra. (**b**–**d**) SERS spectra collected at each measurement point. For clarity, spectra are shifted vertically.

**Fig. 6:**
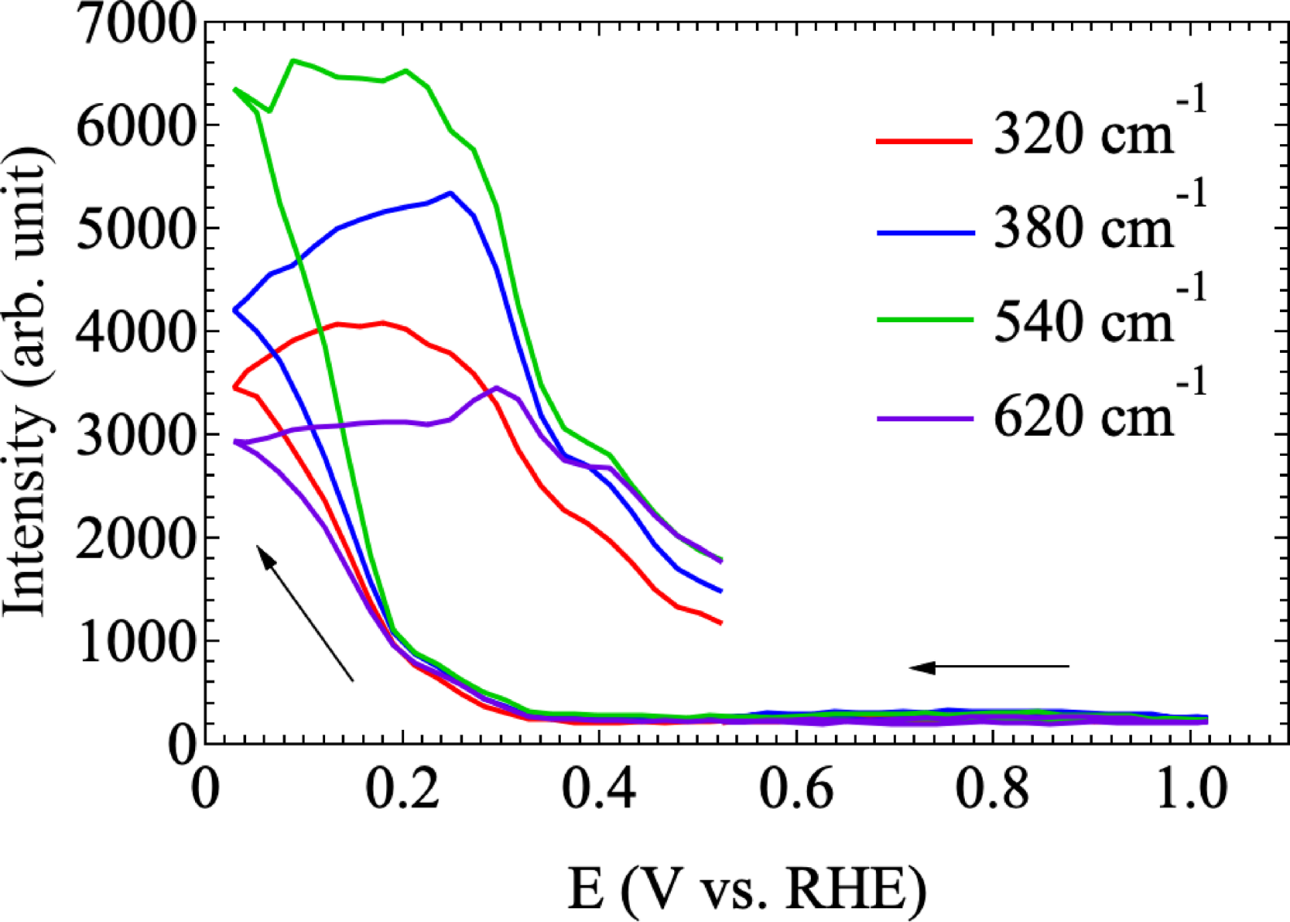
RHE voltage dependence of Raman peaks at 320, 380, 540 and 620 cm^− 1^.

Next, CV of Cu(OAc)_2_ was performed with 4-mercaptobenzoic acid on the surface of the SERS structure to investigate the effect of surface conditions on precipitation. This system was formed by placing 10 µL of 100 µM 4-mercaptobenzoic acid on the electrode and allowing it to dry (step (4) in Fig. 2). Figure 5a shows the first CV cycle of Cu(OAc)_2_ measured under the conditions described above with the 4-mercaptobenzoic acid SAM layer on the GNC electrode. As presented in Fig. 5a, a copper oxidation peak at about 0.50 V and reduction peak at about 0.18 V were observed, along with a new peak at about 0.36 V [corresponding to the point (12)], which appears to be related to 4-mercaptobenzoic acid^46^. SERS measurements were collected during the CV process at the points designated in Fig. 5a. Figure 5b and c show the RHE voltage dependence of SERS spectra during the electrochemical reaction. These SERS spectra are vertically shifted for clarity. First, we focus on the results in Fig. 5b, which confirm that clear SERS spectra were obtained. The peaks at 1056 and 1570 cm^− 1^ were derived from $\:\nu\:(\text{C}-\text{C})$ of the aromatic ring of 4-mercaptobenzoic acid^47 – 49^ The peak at 1360 cm^− 1^ was considered to be due to the stretching COO^−^ vibration [$\:{\nu\:}_{\text{S}}\left({\text{C}\text{O}\text{O}}^{-}\right)$]^47 – 49^ Because a peak at 1360 cm^− 1^ was not observed when 4-mercaptobenzoic acid was not attached to the GNF electrode sensor surface, it is thought that the 4-mercaptobenzoic acid layer on the electrode surface nucleated and grew during CV while also incorporating carbon derived from acetate ions. As shown in Fig. 5b, there was a sharp increase in Raman intensity around the RHE voltage of 0.28 V, corresponding to point (4) and (5) in Fig. 5b. In addition, the SERS spectra during the CV measurements from point (4) to (10) contained new Raman peaks that appeared around 320, 370–380 cm^− 1^ and 520–540 cm^− 1^. According to Debbichi *et al*.^45^ and Michota *et al*.^46^, the Raman peaks at 484–510 and 520–540 cm^− 1^ correspond to the E_g_ and A_1g_ modes of Cu_4_O_3_, respectively. The Raman peak around 370–380 cm^− 1^ and 520–540 cm^− 1^ might be also attributed to disordered CuO_x_/(OH)_y_ species^18^. Considering that the peak shape around 520–540 cm^− 1^ has a shoulder and also induces 484–510 cm^− 1^, it is likely that Cu_4_O_3_ is formed while disordered CuO_x_/(OH)_y_ species and Cu_4_O_3_ are deposited simultaneously. This can be clearly seen from Fig. 6, which shows the RHE potential dependence of each Raman peak derived from 320, 380, 540, and 620 cm^− 1^. The results in Fig. 6 indicate that these Raman peaks appear and decay at approximately the same time. In other words, these new Raman peaks are derived from the transition of Cu valence change from + 1 to + 1.5 and then + 2. It is inferred that the reduction process of Cu, i.e., Cu_2_O $\:\to\:$ Cu_4_O_3_ $\:\to\:$ CuO, can therefore be detected. The Raman peak from Cu_4_O_3_ disappears and that from CuO becomes larger as the reaction progresses to point (16). The maximum intensity of the Raman peak from 4-mercaptobenzoic acid was observed in the range of point (6) to (10), after which it decreased in intensity.

The relationship between the overall electrochemical reaction process and SERS spectra is summarized below. When the electrochemical reaction with the Cu(OAc)_2_ solution proceeds in the reduction direction and 4-mercaptobenzoic acid is present on the GNC electrode as a SAM film, copper precipitation begins at point (1). Because the reduction potential has not yet been reached at point (1), the SERS activity of 4-mercaptobenzoic acid induced by the GNC electrode enables the detection of the SERS spectrum of 4-mercaptobenzoic acid. Subsequently, as copper reduction proceeds, copper nanoparticles are formed on the GNC electrode. The nanoparticles increase the SERS intensity of 4-mercaptobenzoic acid by forming hot spots with the GNC structure as described below. When the CV advances in the direction of progressive copper oxidation, SERS spectra of copper oxides are simultaneously detected with that of 4-mercaptobenzoic acid. In this case, the copper oxides also have a charge transfer effect and emit their own SERS spectra while also exciting the SERS of 4-mercaptobenzoic acid. However, the charge transfer effect is expected to decrease the SERS spectral intensity of 4-mercaptobenzoic acid, because its SERS enhancement effect is small compared to that of the nanoscale gaps between noble metal nanoparticles. The CV curve of the system with a SAM layer (Fig. 5a) showed a new peak around 0.36 V that was lacking from the CV curve of the system without a SAM layer (Fig. 3a). This is because the redox reaction of copper is strongly influenced by the 4-mercaptobenzoic acid that is dispersed in the electrolyte during the electrochemical reaction^44,45^ In other words, 4-mercaptobenzoic acid could bind to copper through COO^−^ bonds not thiol bonds and act as a protective film on the copper surface,^47 – 49^ hindering oxidation. The peak around 0.36 V is therefore considered to indicate a potential energy barrier for further oxidation beyond the influence of this antioxidant film. In the SERS spectra collected after the progression of the CV to higher potential above 0.36 V, i.e., after this potential barrier has been crossed, signals originating from CuO were detected, indicating that CuO formation had occurred. Overall, comparison of the CV curves and SERS spectra with the oxidation process of copper reveals that the oxidation process of Cu to Cu_2_O, Cu_4_O_3_, and finally CuO can be clearly detected in the SERS spectra.

**Fig. 7:**
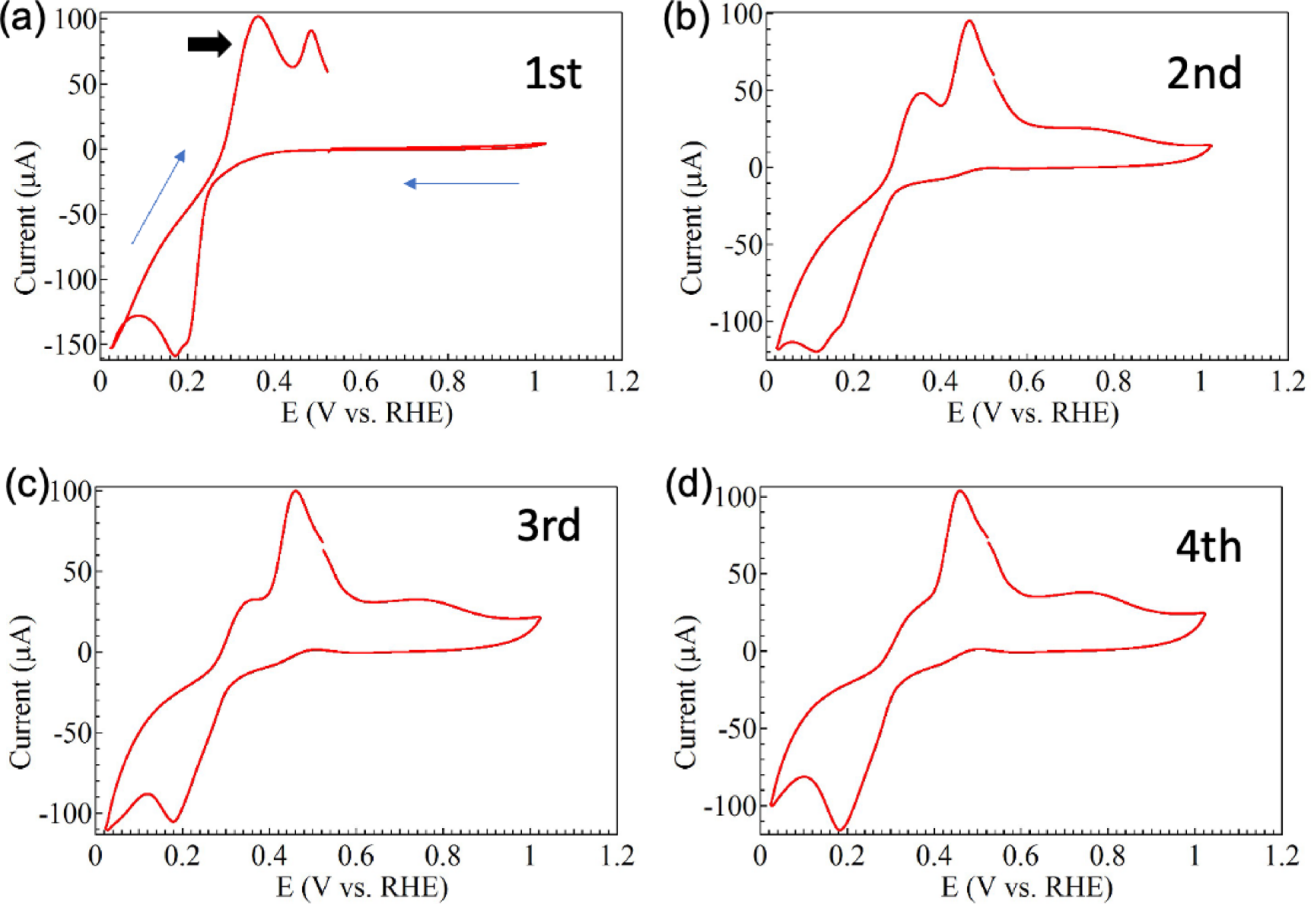
CV curves for the (**a**) first, (**b**) second, (**c**) third, and (**d**) fourth cycle of a GNC electrode coated with 4-mercaptobenzonic acid and immersed in 10 mM copper acetate solution.

**Fig. 8:**
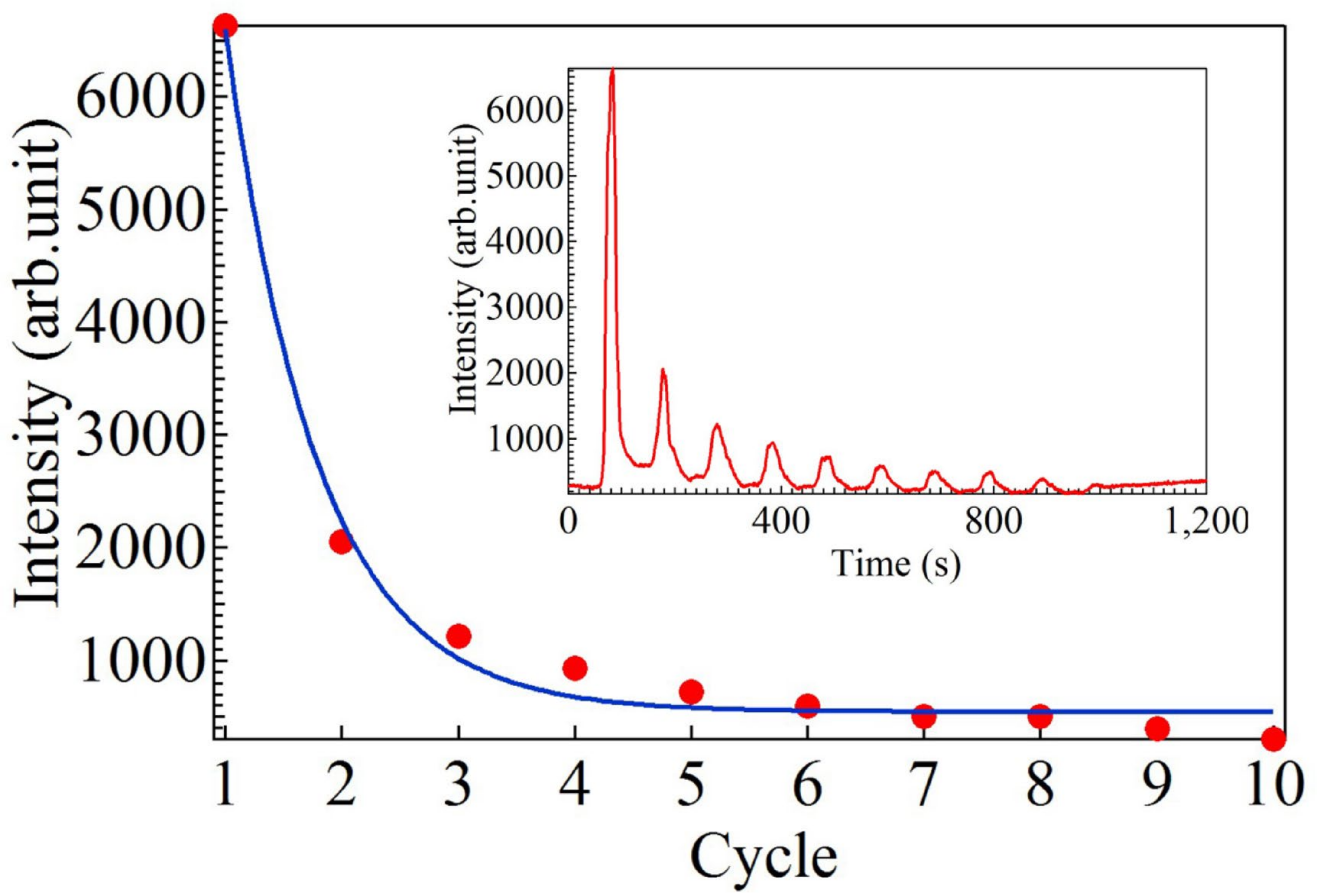
Dependence of the Raman peak intensity at 1600 cm^− 1^ on CV cycle number. The change of Raman peak intensity at 1600 cm^− 1^ over time is shown in the inset. CV cycle number dependence of the Raman peak intensity was fitted using Eq. (8).

Repeated CV measurements confirmed the influence of 4-mercaptobenzoic acid on copper oxidation. The curves for the first, second, third, and fourth CV cycles are shown in Fig. 7a–d. The CV peak that appeared at about 0.36 V in the first cycle clearly decreased in intensity as the number of CV cycles increased. In addition, the peak intensity at about 1600 cm^− 1^ [Raman peak marked with a triangle in Fig. 5b] oscillated and decreased overall as the number of CV cycles increased, as depicted in Fig. 8. This is considered to indicate that the concentration of 4-mercaptobenzoic acid dispersed in the electrolyte decreases during repeated CV cycles. This is because the deposited Cu is not all reduced during oxidation, so the gaps are gradually filled. Because 4-mercaptobenzoic acid is also buried in the copper oxide layer on the electrode, the peak around 0.36 V, which is thought to be derived from the reaction between Cu and 4-mercaptobenzoic acid, gradually decreases in intensity with increasing cycle number. In other words, the formation of hot spots decreases as CV cycle number rises. The SERS intensity strongly depends on the nanoscale gaps between nanoparticles and the number of hot spots present in the laser-irradiated area. The SERS intensity lowers with the decrease of both these contributions. However, it was not possible to distinguish between these contributions in this experiment. Therefore, for simplicity, we assumed a cycle-by-cycle hot-spot decrease rate (*λ*) that included both contributions. The following equation was used to fit the experimental data in Fig. 8.

8 $$\:{I}_{\text{S}\text{E}\text{R}\text{S}}={I}_{0}{e}^{-\lambda\:t}+{I}_{\text{b}\text{g}},$$

where $\:{I}_{\text{S}\text{E}\text{R}\text{S}}$ and $\:{I}_{0}$ are the SERS intensity at cycle number $\:t$ and $\:t=0$, respectively. $\:{I}_{\text{b}\text{g}}$ is back ground intensity. Using Eq. (8), *λ* in Fig. 8  was estimated to be 1.27. *λ* is also considered to be an electrochemical reaction rate constant because the decrease of SERS intensity is equivalent to the progress of an electrochemical reaction.

**Fig. 9:**
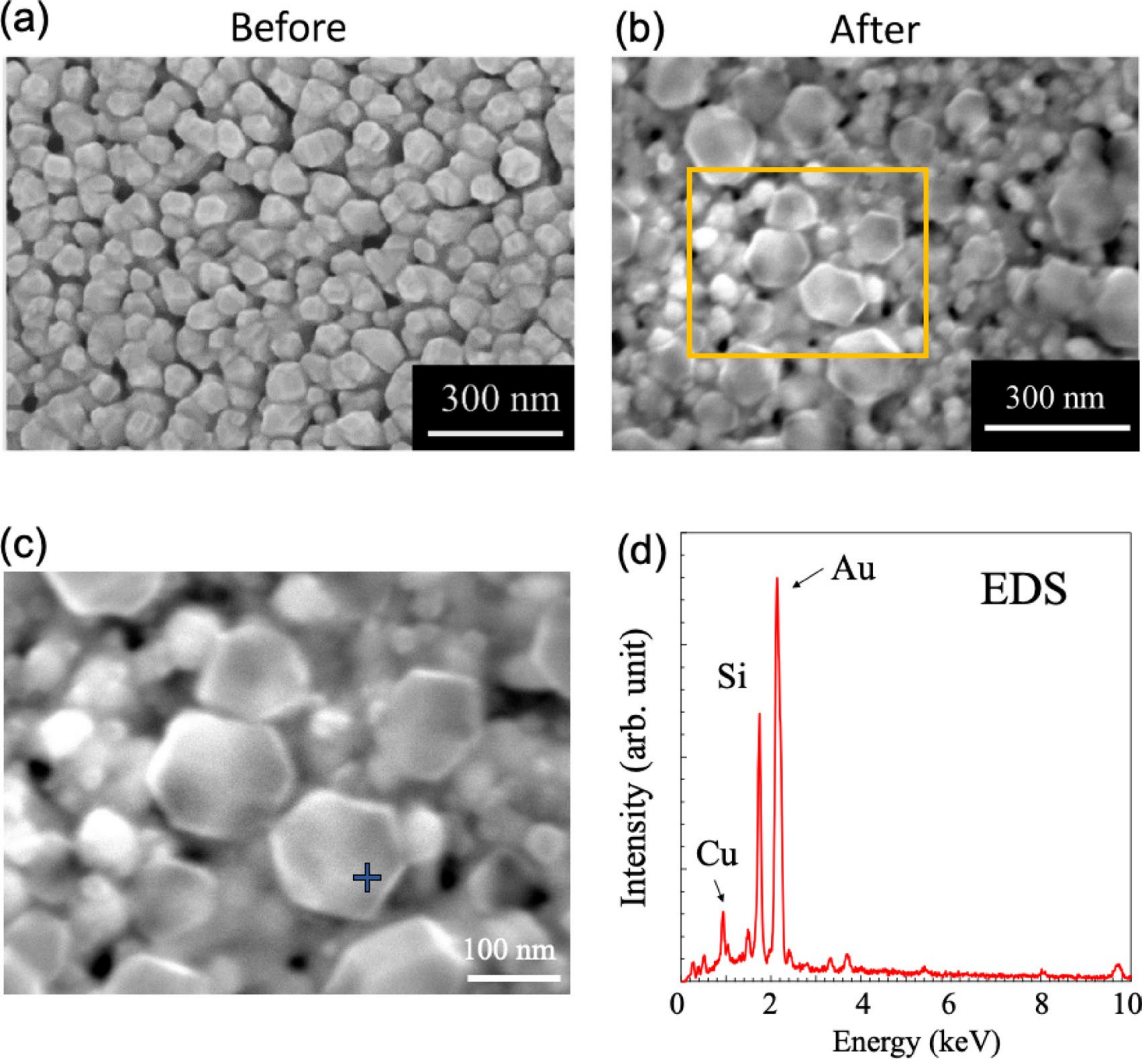
Scanning electron microscopy (SEM) images of the working electrode with GNF structure (**a**) before and (**b**) after CV cycling. (**c**) Magnified SEM image of the area inside the orange open square. (**d**) Energy-dispersive X-ray spectrum measured at the point indicated by a blue cross in (**c**).

To determine if an electrochemical reaction was occurring on the electrode surface, scanning electron microscopy (SEM) observations of the GNC electrode surface before and after the EC-SERS measurements were performed. Figure 9a and b show SEM images of the GNC electrode before and after the CV measurements. Nanoparticles with a polyhedral structure were clearly observed on the GNC electrode after the CV measurements. Energy-dispersive X-ray spectroscopy analysis revealed that the particles on the GNC electrode were copper oxide. Based on previous copper(II) sulfate precipitation studies, it is likely that Cu nanoparticles formed on the SERS structure by growth on the [111] and [100] planes^50,51^.

The addition of 4-mercaptobenzoic acid resulted in the appearance of an antioxidant layer on the product and electrode surface. Using our microchemical system, the effect of this coating on electrochemical reactions was able to be evaluated. Here, to confirm how the properties of mercaptobenzoic acid as a SAM membrane are detected in EC-SERS, the results of EC-SERS in pure water with mercaptobenzoic acid applied to the GNC structure are shown in the Supplementary information^42^ The CV shown in Fig. S1 is the first CV and shows a special CV structure. From the beginning of the CV measurement to the point (1) in Fig. S2, a Raman peak originating from C-C was detected at around 1000 cm^− 1^; the CV shape from the second CV onwards corresponds to the CV shape in Fig. 7d. The peak disappeared as the CV progressed from point (2) to (4) in Fig. S2. Further CV revealed that a Raman peak appeared around 800 cm^− 1^ from point (7) as shown in Fig. S3. This peak was attributed to the C-S bond, indicating that the molecular configuration of the mercaptobenzoic acid binding to the gold structure changed during CV, as shown in Figs. S2 and S3. Thus, our system is expected to become a useful tool for analyzing reactions at solid–liquid interfaces. In addition, this system can be mass produced and easily combined with microfluidic channels. In other words, it is expected to provide a useful evaluation standard chip for researchers and engineers who wish to measure electrochemical reactions and chemical reactions at a surface simultaneously.

**Fig. 10:**
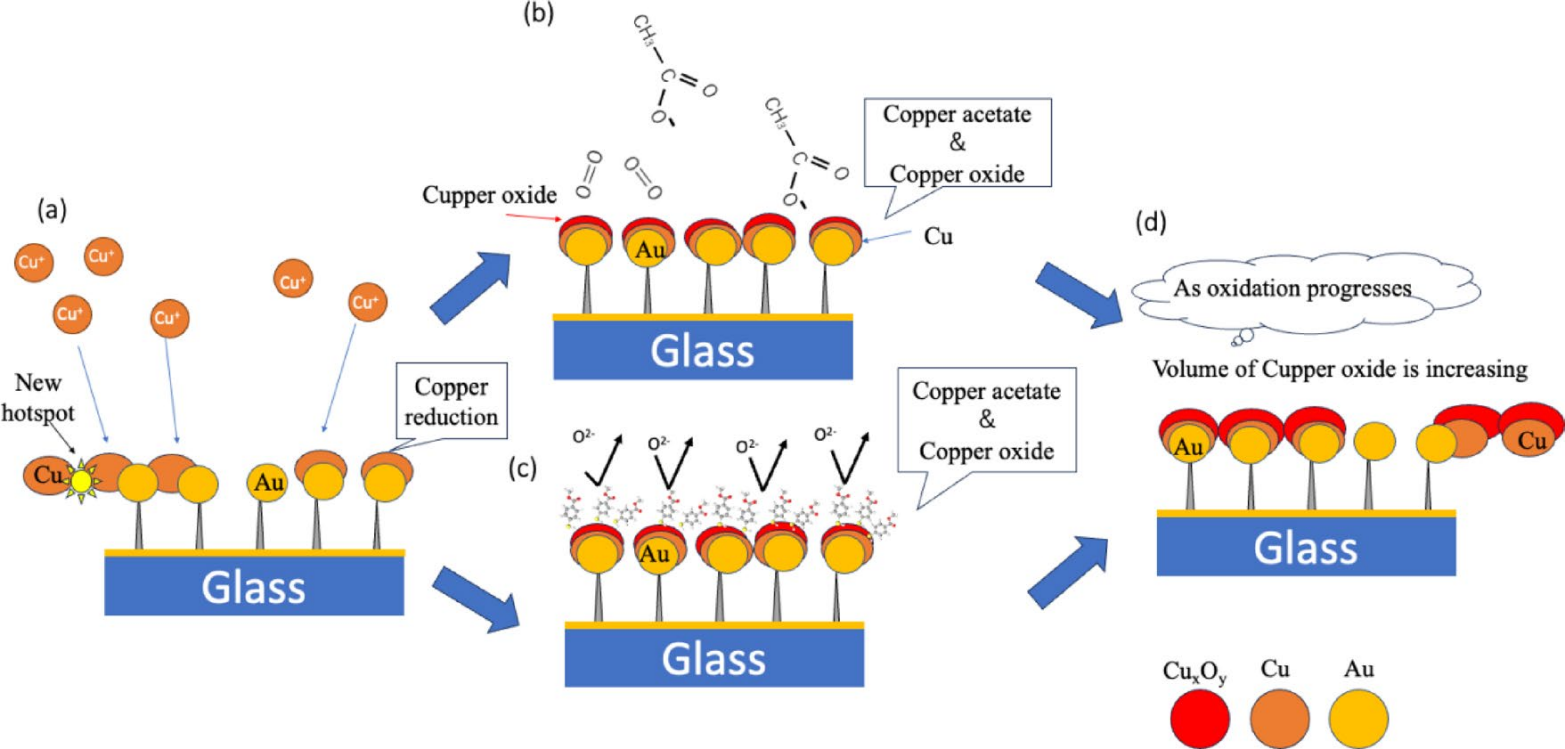
Cross-sectional depiction of the processes observed near the GNC electrode during the CV measurement of copper acetate solution. (**a**) Conceptual diagram of hot spot formation and SERS expression in CV. (**b**) Schematic diagram of ion behavior during CV with copper acetate only. (**c**) Schematic diagram of the electrochemical reaction when the GNC electrode was coated with 4-mercaptobenzonic acid. (**d**) Schematic diagram of copper or copper oxide growth during repeated CV cycling.

In this study, the electrochemical reaction of Cu(OAc)_2_ solution was performed at a GNC electrode with a noble metal nanostructure, so in principle the reaction and copper deposition were induced as schematically illustrated in Fig. 10a. Nano/microscale copper and copper oxide particles formed while being affected by acetate ions and oxygen in the aqueous solution in the case without the SAM film and additives, as shown in Fig. 10b. In the presence of the SAM film, the SAM film acts as an antioxidant, as shown in Fig. 10c. Repeated CV measurements eventually led to the formation and growth of copper or copper oxide particles, which filled the voids in the GNCs, giving the structure shown in Fig. 10d. Such nanostructures may allow for stronger SERS excitation due to light interference effects,^52^ which could be used to track electrochemical reaction dynamics at the solid/liquid interface in operands as in the present study.

The microchemical system can perform electrochemical reactions at very low volumes while SERS measurements are conducted. The system allows the effects of SAM membranes and additives to be tracked at the molecular level. Because the system can be easily combined with microfluidics, it is expected to be useful for monitoring various reaction systems and as a research and development platform for electrochemical reactions.

## Conclusion

We fabricated a microchemical system with a SERS-active working electrode using GNC-structure based on boehmite template. This system can capture changes in molecular structure with high sensitivity while manipulating redox reactions. Our study reveals that the presence or absence of mercaptobenzoic acid significantly alters the electrochemical redox reaction of copper. In other words, this study indicates that chemical reactions such as the redox reaction of copper ions were detectable and evaluated in the presence of adhesions on the electrode surface. The combination of electrochemistry and SERS sensors enables highly sensitive, real-time measurement of chemical reactions occurring on the electrode surface. This technology should be easily transferable to multiple research applications such as fuel cell analysis and metabolic studies using electrical signals. The system can provide an ideal model experimental platform for catalytic reactions, surface coating material functionality and electrode reaction mechanism analysis.

## Electronic supplementary material

Below is the link to the electronic supplementary material.

Supplementary Material 1

## Data Availability

All data generated or analysed during this study are included in this published article. The datasets used and/or analysed during the current study available from the corresponding author on reasonable request.
