# Supplementary material for: Microchemical system for simultaneous measurement of surface-enhanced Raman scattering and electrochemical reactions
Source: Sci Rep. 2025 May 27;15:18574. doi: 10.1038/s41598-025-02647-y (PMC12116894; doi:10.1038/s41598-025-02647-y)
Supplement: Supplementary file 1 — Supplementary Material 1 [file 41598_2025_2647_MOESM1_ESM.docx]

[Supplementary Information]

**Microchemical system for simultaneous measurement of surface-enhanced Raman scattering and electrochemical reactions**

Shunya Saegusa ^1^, Masayuki Naya ^2^, Takao Fukuoka ^3^, Miyuki Tabata ^4^, Koji Sumitomo ^5^, Akinobu Yamaguchi ^6*^

^1^ Laboratory of Advanced Science and Technology for Industry, University of Hyogo, Ako-gun, Hyogo 678-1205, JAPAN

^2^ Faculty of Science and Technology, Keio University, Yokohama, 223-0061 JAPAN

^3^ Department of Micro Engineering, Kyoto University, Kyoto Daigaku-Katsura, Nishikyo-ku, Kyoto 615-8540, Japan

^4^ Graduate School of Bio-Applications and Systems Engineering, Tokyo University of Agriculture and Technology, Naka-cho, Koganei, Tokyo 184-8588, Japan

^5^ Graduate School of Engineering, University of Hyogo, Shosha, Himeji, Hyogo 671-2280, Japan

^6^ Department of Electrical, Electronic and Communications Engineering, Faculty of Science and Engineering, Toyo University, 2100 Kujirai, Kawagoe, Saitama 350-8585 Japan

*Contact address: [yamaguchi054@toyo.jp](mailto:yamaguchi054@toyo.jp) (A. Y.)

**Electrochemical surface-enhanced Raman scattering (EC-SERS) of 4-mercaptobenzoic acid film on a gold nanofève (GNF)-SERS-active structure electrode (pure water)**

Cyclic voltammetry measurements were carried out on 4-mercaptobenzoic acid (MBA) on GNF-SERS-active structure electrodes in order to capture changes in Raman spectra due to electrochemical chemical reactions and desorption of molecules by controlling the electrochemical potential on the GNF-SERS-active structure electrode. The experiment was performed as follows. First, 10 µL of MBA with the concentration of 100 µM was dropped onto the SERS-active working electrode and allowed to dry. Then, 40 µL of pure water was added and SERS spectral measurements were performed while cyclic voltammetry (CV) was measured. Figure S1 shows the CV result of the first cycle; a sharp oxidation peak was observed at around 1.5 V. This behavior was observed in the first cycle only. Subsequent CV was consistent with the result shown in Fig. 7(d). Now, we turn our attention to the CV of the first cycle. After starting the CV measurement, the Raman spectrum in the inset of Fig. S1 was obtained at the application of 0. 7 V. Basically, this Raman spectrum was observed during the voltage sweep to the point (1) in Fig. S2. This Raman peak is originated from the C-C bond around 1000 cm^-1^ of MBA. With further voltage sweeps, this peak disappears as shown in Fig. S2. This result suggests that the MBA was planarly attached to the benzene ring on the surface of Au on the GNF-structure, and that the MBA was desorbed from the GNF-SERS-active electrode by the application of voltage. On the other hand, CV was performed and the C-S bond peak appeared rapidly at the reduction peak position when the voltage reached around 1 V [corresponding to point (7)] as shown in Fig. S3. This result could be due to the reattachment of MBA onto the Au surface via the thiol group side. As described above, the process of MBA desorption was successfully detected, indicating that changes in the sensor surface state can be traced.

　The results show that MBA coats the gold surface via thiol bonds when CV is performed, providing evidence for thiol coverage with regard to the experimental results reported in the paper.


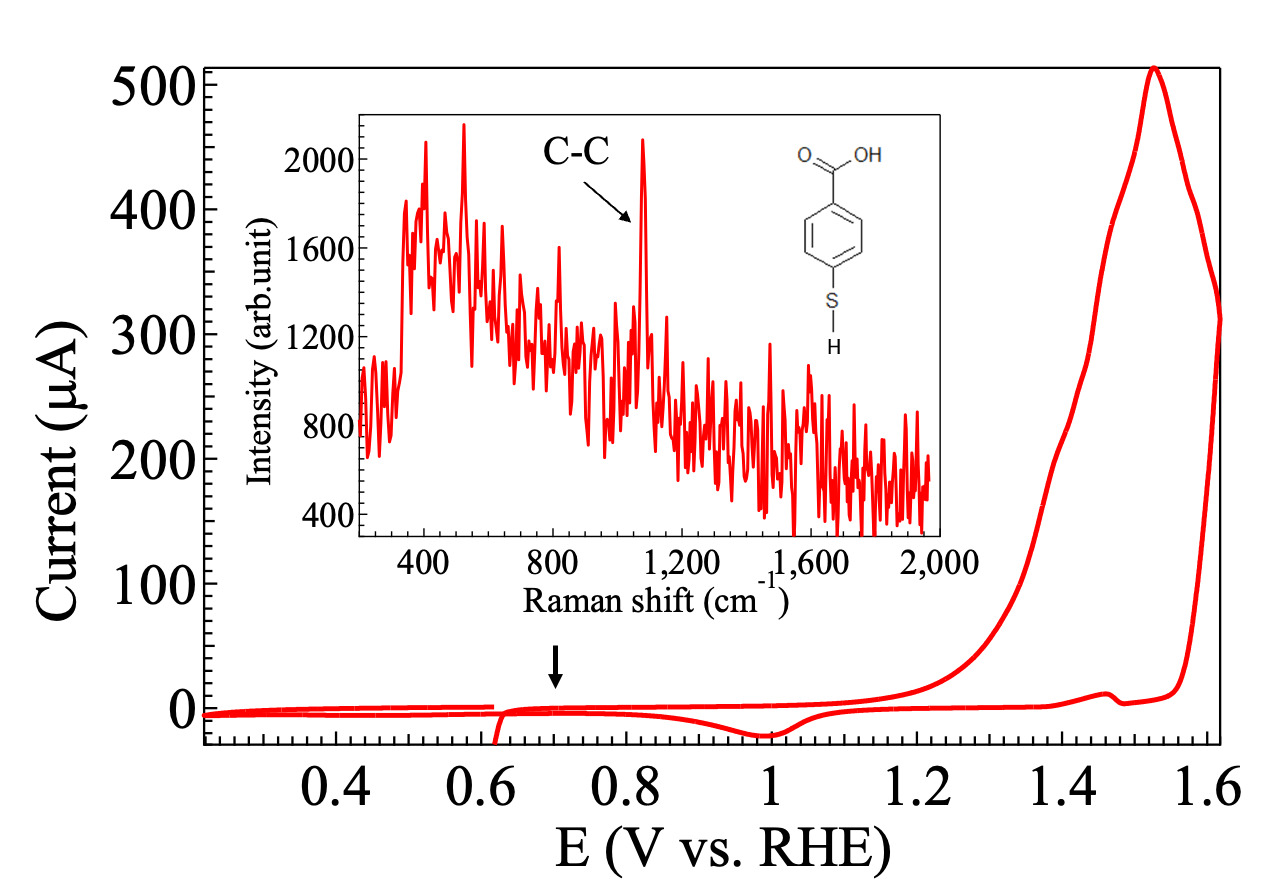


Figure S1 First cycle of CV for MBA on GNF-electrode in 40 µL pure water. This behaviour was observed in the first cycle only. Subsequent CV was consistent with the result shown in Fig. 7(d). The inset shows the SERS spectrum of MBA at 0.7 V.


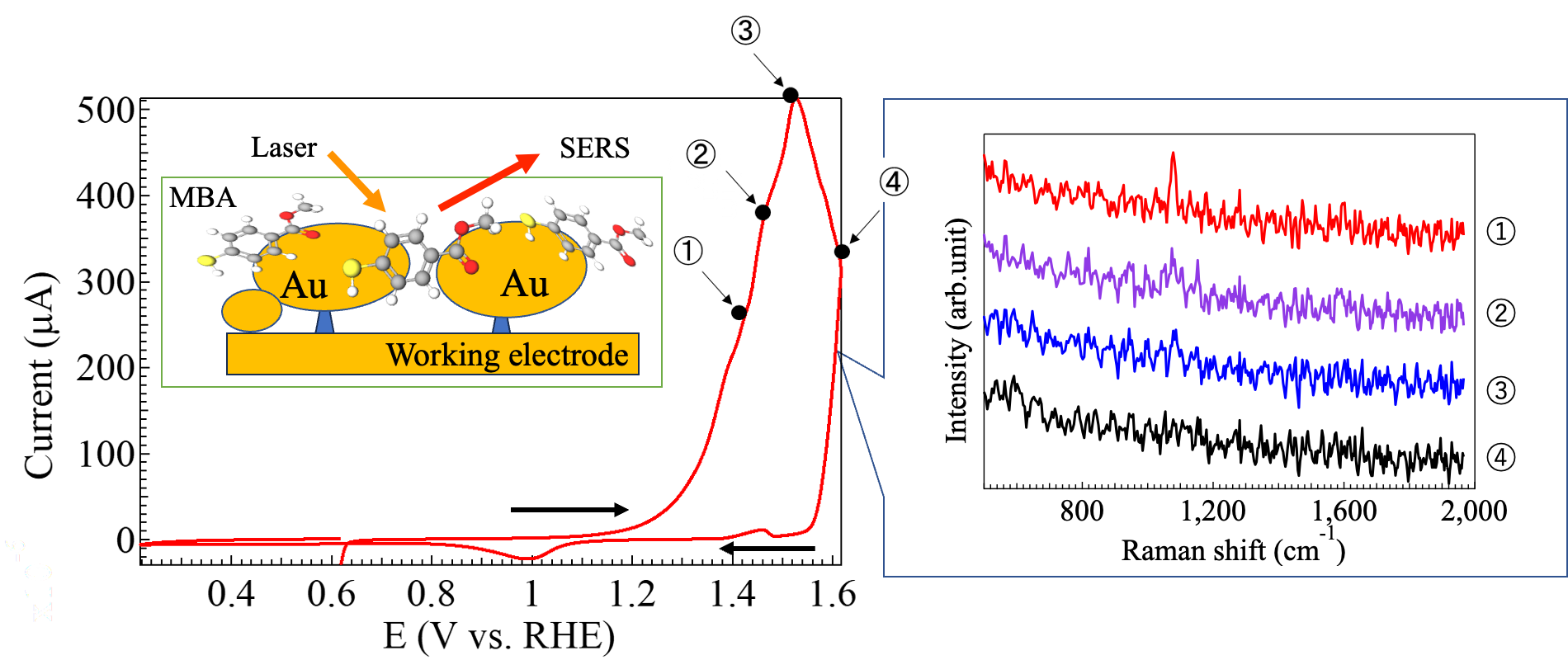


Figure S2 EC-SERS measurement results obtained at points from (1) to (4). The inset shows schematic of EC-SERS at the point (1).


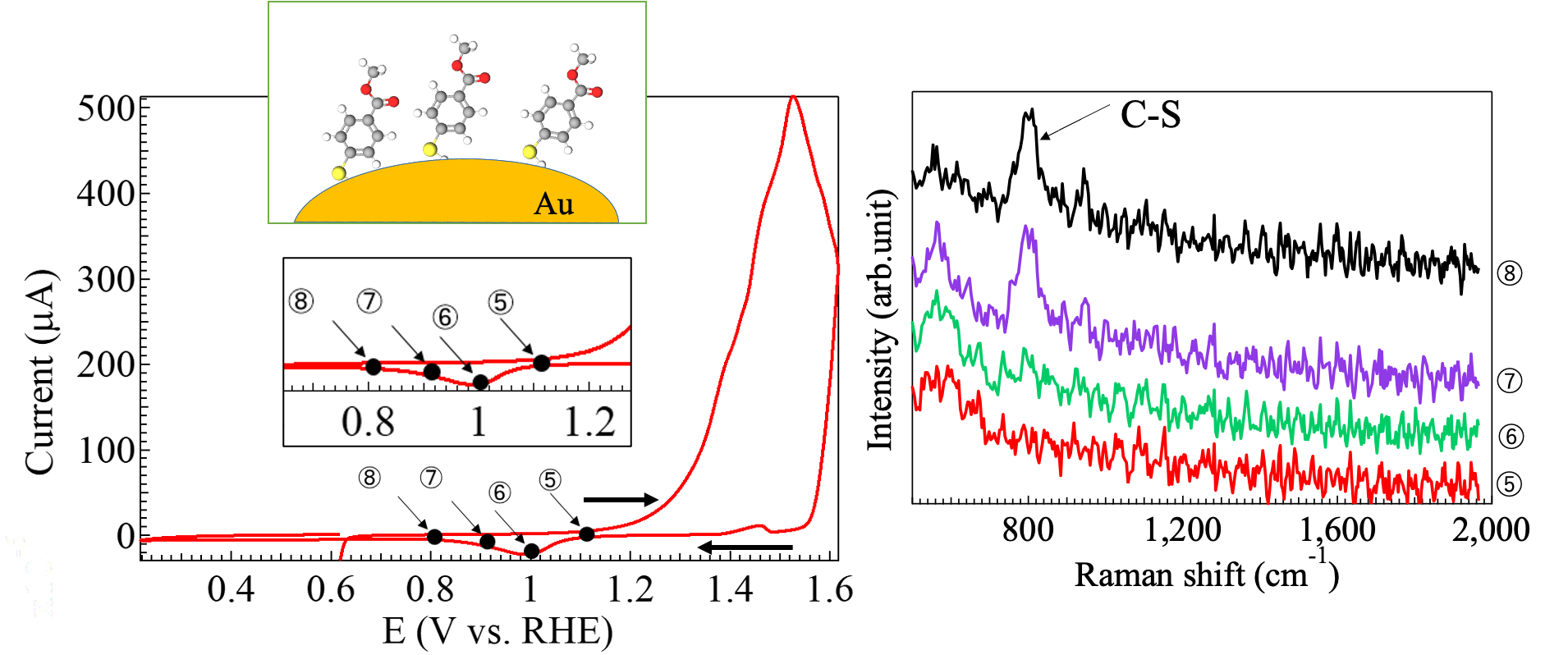


Figure S3 EC-SERS measurement results obtained at points from (5) to (8). The magnified CV and the schematic diagram of thiol-linked molecular arrangement are shown in the inset.
